# The History, Diagnosis and Treatment of Fibrinous Coagula of the Heart

**Published:** 1850-05

**Authors:** David W. Yandell

**Affiliations:** Louisville, Ky., Attending Physician for the Faculty at the Louisville Marine Hospital


### WESTERN JOURNAL

OF

MEDICINE AND SURGERY.

---

MAY, 1850.

---

Art. 1.—*The History, Diagnosis and Treatment of Fibrinous Coagula of the Heart.* By David W. Yandell, M. D., of Louisville, Ky., Attending Physician for the Faculty at the Louisville Marine Hospital.

### ARTICLE I.—Introductory.

The disease of which this memoir gives an account is much less generally understood than any of the other diseases to which the heart is subject. Recent pathological discoveries have revealed it to be of much more common occurrence than has hitherto been believed. While the pathology of this disease is definitely and satisfactorily understood, its causes, diagnosis, and particularly it treatment, are still involved in doubt and uncertainty.—

There is no full and complete history of it in the English language. It will be my object to supply, as far as is in my power, this deficiency, and "to furnish," so far as may be possible, "such knowledge of this disease as is essential to its successful treatment. This knowledge ought at all times to be ready for use and application. The necessity for this use and application, whenever it occurs, is immediate, urgent, and pressing; it admits of no delay; there is no time for deliberate inquiry and consultation. The physician, in many, perhaps in most cases, is obliged to act upon his own single responsibility; it is his duty to be fully prepared, in advance of the emergency, to act promptly and aright."

### ARTICLE II.—Lesions.

*Preliminary.*—I shall assume that the researches of Testa, Burns, Laennec, Corvisart, Bouillaud, Kreysig, Andral, Hope, Hasse, and other pathologists have established the fact, that there are some fibrinous coagula formed within the heart, during and after dissolution, and others formed for a longer or shorter period anterior to it, presenting various degrees of organization, and they are the cause of several well marked symptoms during life.

Long before any definite ideas of the true nature of the concretions in question were entertained, it was known that they were of more frequent occurrence in the right than in the left side of the heart. M. Bouillaud asserts that they are also more frequent in the auricles than in the ventricles. The principal cause of this difference is, that the blood is more easily retarded and rendered stagnant in the right cavities, and that it is in them especially that it accumulates during the last period of life, and after death. Bouillaud believes that other causes also may explain the circumstance: such as the frequency of phlebitis, which is sometimes propagated into the right cavities; perhaps, a more marked tendency to coagulation in the venous, than in the arterial blood, &c., (Traité ii., p. 711, ed. 1841.)

*Anatomical characters.*—Hasse acknowledges two types of the blood coagula, the *unorganized*, and *more completely organized*, the distinctions between which are essential and obvious; there are, however, he remarks, so many transition forms, that it is often extremely difficult to come to a definite conclusion.* I believe, however, with Dr. Hope, that there is a third variety, which is possessed of equally distinctive anatomical differences, and which I shall include under the name of "*slightly organized polypi*," being the term given to it by that distinguished author. (Hope. Diseases of the Heart, p. 483. American edition.)

I. *Unorganized Polypi.*—Polypi formed after death, or during the last moments of life, are concretions of fibrin, identical with the simple fibrinous clot, so common in robust individuals. They are of a smooth, shining surface, yellow, pellucid, of the elasticity of jelly—particularly in dropsical subjects, or when the blood is very serous, and are "fashioned to the shape of the heart's cavities, especially at the origin of the pulmonary artery, where they often appear moulded with a faint impression of the valves. The gelatino-fibrous mass occasionally covers a clot of coagulable blood, in the same proportion as the buffy coat, the crassamentum in blood drawn from a vein; it, however, frequently constitutes the bulk of the concrement, and contains only a few portions striated or dotted with blood red, (Hasse.) Polypi of this description are more common and of greater volume on the right side of the heart, than on the left, are often found "proceeding, with flattened ends, into the large vessels," and

*The microscopic and chemical relations of coagulated fibrin will be found described in Henle's Annual Report, in Henle & Pleuffer's Journal, 1843, p. 168.

present no trace of internal organization and structural arrangement. The amount of these concretions is variable· They seldom, however, fill the cavities, and never adhere to their parietes. Bouillaud, it is true, has reported cases where the coagula in the different cavities of the heart amounted to twelve ounces, but it is necessary to state that a large proportion of them were formed after death. These characters are, in the opinion of Dr. Hope, sufficient to distinguish them from polypi formed sometime previous to death.

II. *Slightly Organized Polypi.*—It is a well known fact, that fibrin separated from the blood and become concrete in a living organ, preserves its vitality and is susceptible of organization in an equal degree with inflammatory lymph.

Polypi formed sometime before death, in which this organization has commenced, are of a much firmer consistence; more opaque, and less charged with serum; their fibrous texture is more distinct; they are often arranged in concentric layers; their color, instead of being uniformly whitish or yellowish, has, in parts, a pale flesh tint, sometimes slightly violet, from incipient vascularity; they are found more frequently on the left side of the heart than recent polypi are; and they adhere more or less firmly to the walls of the heart, from which it is scarcely possible to draw them away in a single piece, as the extremities remain attached under the columnæ carneæ.—The medium of adhesion is often a filamentous tissue, the rupture of which leaves a roughness both on the lining membrane of the heart and on the surface of the polypus. The surface also presents spots of blood penetrating more or less deeply, and sometimes ramifying inwards, as if to form vessels for the purpose of organizing the mass. Some of these polypi contain pus in the center; sometimes pure, at others, curdy or sanious—precisely what we so commonly see within coagula formed by phlebitis.* (Hope on Diseases of the Heart, p. 483, and Hope's Morbid Anatomy, figs., 204 and 240.)

Dr. Hope considers the *globular vegetations* of M. Laennec, mere varieties of these suppurating polypous concretions. " They present themselves under the form of irregularly spheroid or ovoid balls or cysts, the size of which varies from that of a pea to that of a pigeon's egg. The cysts are smooth externally; and their walls, which scarcely exceed half a line in thickness, are composed of an organized substance somewhat firmer than the white of a hard-boiled egg, and resembling in opacity the oldest polypous concretions. The internal surface of the cyst is less smooth than its exterior, and appears formed of a softer substance, which sometimes gradually degenerates, in the direction from without to within, into a matter similar to the contents of the cyst. These contents, in the cysts which there is reason to believe the most recent, are bloody; in the older, they are like lees of wine,

---

*Many writers, and especially M. Legroux, think this pus a product of inflammation of the concretion which contains it. "An inflammatory movement," says M. Legroux, "shows itself in the concretion. * * * * * * It softens in the centre, becomes granulated, passes to the sanious, then to the purulent state: subsequently, the pus is absorbed, and there only remain the exterior layers of the concretion, which have resisted the softening, and which form the walls of the abscess or the cyst. (Legroux. Inaugural Dissertation, p. 35.) Dr. Hope is of the same opinion. Bouillaud, on the other hand, holds the following language on this point: "I think that such is not the ordinary origin of the pus which is found in concretions; this *product* appears to me to have been either secreted in the cavity of the heart, or to have been transported thither by absorption, and then to have occasioned the formation of a coagulum which has entirely enveloped it. At the period when pus in the center of a concretion is most frequently met with, the concretion offers scarcely the rudiments of organization, and one can hardly conceive how, in this stage, it could undergo an inflammation characterized by purulent secretion. I do not pretend to say, however, that when once well organized, sanguineous concretions may not inflame and suppurate. Nevertheless, this is not, if I do not mistake, a very common occurrence, (Traité, II, p. 610, edition 1841.

and in the oldest, they are puriform. The cyst adheres by a pedicle, which, according to M. Laennec, is of more recent formation than the cyst itself, being more translucent, and in a less advanced state of organization. The pedicle is interlaced among the columnæ carneæ, and united more or less firmly with the internal membrane.—The most common situation of these bodies, and where I have frequently found them, is about the apex of the ventricles. I am not aware that they are ever found in the great vessels; I have never seen them there," (Hope, p. 484.)

III. *More completely organized Polypi.*—When the polypi are in a more advanced state of organization they adhere by a true cellular tissue to the parts with which they are in contact, (Bouillaud.) They may be dated as far back as several months prior to the death of the patient, * * * * are completely opaque, like paste or cheese, exactly resemble the oldest fibrinous layers of false aneurisms, and adhere so firmly to the walls of the heart, that they cannot be detached without scraping with the scalpel, and sometimes not without removing the internal membrane, (Hope.) Engrafted thus, upon the living tissues, vessels shoot through them, they harden and assume a striking resemblance to certain fibrous polypi, to fungous tumors or vegetations, (Bouillaud.)*

*Concerning the connexion which exists between fibrinous coagula and the origin of certain lesions of the valves of the heart, (the adhesion of their angles and tendons with each other,) MM. Bouillaud and Legroux entertain the same opinion. M. Legroux has frequently seen fibrinous concretions interlaced with the tendons of the valves, in such a manner that these no longer formed a simple curtain, (the division into tendons not taking place until near the columnæ carneæ.) He also has no doubt that these concretions are the principal means of the union of the tendons, in cases where the lengthened valves no longer form a simple opening but a canal of greater or less size, (Legroux, op. cit., p. 35.) Both these writers further believe that in order for these concretions to contract intimate and firm adhesions with the valves on which they are deposited, it is necessary that they be formed under the influ-

Polypi, at their several degrees of organization, may undergo various changes. Some of them have been already mentioned. Bouillaud, in the Journal Complimentaire du Dictionnaire des Sciences Medicales, details a case wherein the concretion had been converted into a substance which bore a strong resemblance to encephaloid. M. Velpeau and others have since published similar cases. M. Dupuytren also met with examples of this kind.—He asserts "that the pretended carcinoma of the blood is but puriform matter developed by animal heat in the concretions." M. Bouillaud believes that polypi of "recent formation and small volume may be dissolved." In certain cases, also, he thinks it probable that fibrinous coagula of the heart, of small volume, are expelled into the circulation.*

---

ence of inflammation; in such a case it is difficult to decide if these concretions are developed at the expense of the blood contained in the heart, or are the result of the secretion of an inflamed tissue. They both believe that the concretions which are firmly adherent, and susceptible of being transformed into vegetations, are the result of *an inflammatory secretion which adheres to the membrane at the moment of its formation*, (Bouillaud.)

* A very novel hypothesis has recently been advanced by the distinguished Professor Meigs, which relates so directly to the present division of our subject, that we cannot refrain from giving it a passing notice.

At one of the late meetings of the Philadelphia College of Physicians, it appears that Professor Miegs accounted for the removal of fibrinous concretions of the heart, and subsequently revealed the true origin of tuberculosis. With the highest respect for the talents, and greatest deference for the age of the learned gentleman, this, we think, is a work of much too great magnitude to have been despatched so summarily.

The train of reasoning by which Professor Meigs, on the occasion alluded to, arrived at this discovery (?) is quite as novel as the discovery itself. We give it *in extenso*, as an example of imaginative logic. It runs thus: Dr. M. was once present at a postmortem examination of a body in which there existed a large coagulum of blood in the right auricle of the heart; it nearly filled the cavity; the upper portion was rounded, smooth and polished; the lower portion was prolonged, and penetrated through the auriculo ventricular foramen, into the right ventricle, presenting there a ragged appearance, as though it had been gnawed by a

The fibrous, cartilaginous and cretacious transformations, which they are sometimes found to have undergone, are of very rare occurrence, and Grisolle affirms are never seen, except in cases where the concretion has depended on inflammation of the endocardium, and was accompanied by a pseudo-membranous exudation. It is needless to say that transformations of this nature may and often do become the origin of serious disease of the centre of the circulation. M. Grisolle has frequently seen adhesions of the valves, narrowing of the orifices, &c.,

---

rodent, it having been evidently threshed and beaten by the cordæ tendinæ of the heart, into minute particles. [This, we have little doubt, is one of the processes by which the heart gets rid of recently formed coagula.] These small, comminuted fragments, into which a portion of the clot had been reduced, must have been carried by the torrent of blood passing from the heart, through the pulmonary artery, into the lungs, and there become arrested, when they reached the commencement of the capillaries. Here, being left in contact with the endangium, the common lining membrane of the whole arterial system, the questions suggested themselves to Dr. Meigs, what, then, becomes of them—what office do they then perform? He thus answers them: They—the fragments—may become compressed, but they cannot be driven onward by the action of the blood behind them: nor can they become either dissolved, absorbed, or decomposed; they consequently must remain, and forming special sacs, by gradually pressing out, laterally, that portion of the endangium with which they are in contact, into the adjacent cellular texture, *they become tubercles*. Years may pass by, and still find them in their quiescent state, but should inflammation occur in the lungs, from any cause, then do these arrested and encysted coagula become the cause of extensive disorganization in the surrounding tissues and of death.

We can very willingly credit the manner in which the heart overcomes, and eventually removes the obstacles opposed to its free action, in the form of coagula in its cavities or at its orifices, but that these infinitessimal portions of fibrin, no matter where arrested, whether in the capillaries of the lungs, mesentery, or brain, finally become tubercles, or have any real or natural connection with the formation of these bodies, we confess ourselves wholly at a loss to conceive. It is clear to our mind that we might, with as much reason and equal facility, establish that these "comminuted fragments" may, on sufficient provocation, be converted into any other of the malignant growths, into hydatids, biliary, renal, or urinary calculi, or, if allowed full scope, produce spasm, palsy, dysentery, prolapsus uteri, conjunctivitis, or blennorrhagia.

produced by degenerated, or, more properly, differently transformed fibrinous concretions.

### ARTICLE III.—Causes and Formation of Fibrinous Coagula.

The causes which produce fibrinous concretions of the heart are *mechanical* or *vital.*

I. When fibrinous coagula result from mechanical retardation, and consequent stagnation of the blood, they occur under circumstances the most favorable to that stagnation; namely, during the last hours or days of waning life, in all diseases—especially chronic diseases which have occasioned cachexy, emaciation, extreme debility, or which have been accompanied by any considerable obstacle to the general circulation; for instance, dilatation with attenuation, softening, or great valvular disease of the heart. Under these circumstances, so retarded is the circulation, that blood will scarcely flow from veins opened by the lancet, and it is sometimes actually coagulated in them.* Dr. Hope mentions a number of cases of phthisis, in which this coagulation took place in the femoral veins, and caused edema of one or both extremities. That stagnation alone suffices to cause coagulation is a fact too familiarly known to require demonstration. We see it, out of the body, in blood drawn by the lancet; we see it exemplified within the body, by the fibrinous concretions that fill up false aneurisms; the operations for this disease, moreover, has, for its basis, the coagulation in question.† We see it in the thick and demi-concrete blood which follows venesection in the last moments of life, in the diseases of the valves and orifices of the heart, which greatly embarrass the circulation, &c.

Dr. Hope believes the adhesion of coagula from stag-

---

* Hope, p. 485. * Hope, loc. cit.

nation, to be occasioned by the irritating action of the bodies themselves on the walls of the heart; whence there results an exudation of lymph on the latter, which forms the agglutinating medium. He once saw a case in which loose coagula existed in most parts of the venous system —but, in the vena portæ, they were adherent wherever large trunks, subdividing into others, too small to admit them, had arrested their progress. (Had Dr. Meigs seen this case, the much mooted question of the origin of tuberculosis would have been resolved years ago.)

II. The vital or chemical causes are of three kinds: First, That which depends on a primitive or consecutive inflammation of the lining membrane of the heart, which concretes the blood at its surface. Second, The introduction of pus and different foreign substances, as mercury, mineral acids, &c., into the torrent of the circulation, thereby rendering the blood more coagulable. Third, To Burserius belongs the merit of having first made known the fact that the blood possesses an especial tendency to coagulate in the course of certain inflammatory diseases, particularly in pneumonia and acute articular rheumatism.* M. Grisolle, in his Pathologie Interne, suggests that, in these cases, the fibrinous concretions are formed by the same mechanism as the *couenne pleurétique.*

The well known fact that one of the first effects of inflammation of the coats of an artery or vein, is to dispose the blood within the vessel to coagulate at the inflamed portion, warrants the belief that the same thing may occur equally within the heart, when its interior is inflamed. The cases of fatal acute endocarditis, detailed by M. Bouillaud, in which there were concretions evidently formed sometime anterior to death, afford to the mind of Dr. Hope strong evidence that such is actually the case.

Pus, mineral acids, &c., introduced into the circulation,

* Grisolle. Traité de la Pneumonie, p. 75.

are other well known causes of coagulation of the blood by chemical or vital influences.

Relative to the third division of the vital or chemical causes, the general inflammatory condition of the blood, M. Bouillaud thus writes: "All pure inflammations, attended by violent fever, and when the blood drawn from a vein presents a good, firm, elastic, resistant, buffy coat, constitute a real predisposition to certain fibrinous concretions of the heart, which have then a great resemblance to the inflammatory buff." After referring to cases contained in his work on diseases of the heart, and to a memoir published by him in the Journal l'Experience, he continues, "it will be seen that in most of the instances in which the fibrinous concretions did not proceed from a mere embarrassment of the circulation, they accompanied either an idiopathic inflammation of the heart, or an inflammation of another organ, which reacted smartly on the heart, as well as on the whole circulatory system and mass of blood," (Traité, ii., p. 716.)

While Dr. Hope thinks this doctrine far from improbable, he conceives that it will "require for its establishment a greater number of cases than are recorded in the works of M. Bouillaud; for we must not," he says, "come too hastily to the conclusion in question, when we consider how frequent are acute inflammations, and how comparatively rare is their termination in polypus." The testimony recorded in the works of MM. Bouillaud, Grisolle, and Legroux, is, in my mind, conclusive as to the truth of this doctrine.

This tendency in the blood to coagulate in the phlegmasia is, as we have already observed, more remarkable in some diseases than in others, in pleuro-pneumonia, pneumonia, and acute articular rheumatism, for example. "In every patient who, during a period of three years, has died in our wards, of pure acute pleuro-pneumonia, we have found in the heart and great vessels fibrinous coagula evidently formed in part before death. We have also met with similar concretions in other subjects, victims to other purely inflammatory diseases," (Bouillaud.)

So striking is this disposition in the blood to coagulate in this particular form of disease—pleuro-pneumonia—that M. Bouillaud, with his characteristic confidence, lays down, as a law of pathological anatomy, "*that fibrinous concretions are constantly present in persons who die of a pure acute pleuro-pneumonia, which is well characterized, and has reached the second or third degree*, (Op. cit., p. 718.) The same author reports a case of fibrinous coagula in an individual affected with grangrene of one of the inferior extremities.

The second and third cases, in the dissertation of M. Legroux, are examples of concretions occurring in spontaneous gangrene. In the Gazette Médicale, for the 25th of December, 1832, it will be found stated that "in this form of gangrene, the blood has a peculiar tendency to coagulation throughout the whole extent of the organism." On this point we do not hazard an opinion.

From all that precedes, I think, with Laennec, that we may deduce the following conclusions:

1. The remora of the blood, in consequence of obstruction to its course, suffices in itself to produce coagulation, and to determine the formation of a coagulum of organizable fibrin. Every cause capable of occasioning this remora, particularly mechanical obstruction to the circulation, and repeated and prolonged faintings, appears to me sufficient to produce this effect.

2. The coagulation of the blood in the vessels seems to produce, in some cases, particularly in the veins, a true inflammation, accompanied by the formation of a false membrane.

3. It appears certain, that occasionally, and especially in the veins, where the circulation is slow, an inflammation of the inner coat of these, may occasion coagulation of the blood, in the vicinity of the lymph effused by the inflammation.

4. Pus absorbed in great quantity by a vein, may in several ways affect the concretion of blood—by rendering it less liquid from simple admixture,—by coagulating it by a chemico-vital action,—and by exciting inflammation of the containing vessel, (Laennec.)

And we may add, (5thly,) a *general* inflammatory condition of the blood, may, particularly in certain acute diseases, give rise to the formation of fibrinous coagula.

### ARTICLE IV.—Signs and Diagnosis of Fibrinous Coagula of the Heart.

Pasta,* Morgagni, Kerkring, and many other writers of the two last centuries, denied that it was possible for the blood to become concrete during life, and consequently affirmed that it was incorrect to attribute to this cause any appreciable symptoms whatever. Others, again, attributed to concretions of the heart a number of lesions both of the circulation and respiration, which have since been determined to depend on other and organic disease of this organ. We are told that J. J. Rousseau repaired to Montpelier to be treated for polypus of the heart, a journey, as M. Bouillaud observes, which the illustrious melancholic could not possibly have performed on foot, had he been really laboring under the disease, the idea of which so deeply troubled his ardent imagination.

I. Fibrinous coagula, of small volume, offering no obstacle either to the play of the valves or cardiac circulation, give rise to no perceptible functional disturbance.—Under different circumstances, they produce various symptoms—the more important of which we propose to enumerate.

---

* The opinions of J. Pasta will be found contained in two letters published by his kinsman, A. Pasta, and entitled: "*De motu sanguinis postmortem et de cordis polypo in dubio revocato,*" published in 1737.

One of the earliest, most evident and necessary effects of fibrinous concretions of the heart, is to cause a greater or less obstacle to the circulation, according to their volume and situation. Dr. Hope has generally found those filling up an auricle, produce this effect in a greater degree than any others—probably, he suggests, because the auricle, from having less contractile power to expel the stagnating blood, gets more completely charged with concretions; and partly, also,because auricular-polypi usually send off prolongations or projections into the orifices, which not only impede the action of the valves, but also choke up the passage. Those concretions which distend the auricular appendage, produce no disturbance, while those which occupy one of the orifices of the heart, will give rise to all the accidents of organic narrowing. When the concretion attains sufficient size to nearly obliterate the cavities or orifices of the heart, and completely intercept the course of the blood, death rapidly supervenes in a state of suffocation or syncope.

When the coagula are of considerable volume and distend the cavities of the heart, the symptoms are those of great embarrassment in the circulation. The pulse is small, unequal, and intermittent; the face is livid; the extremities cold; in most cases, there is nausea and vomiting of all ingesta; an intolerable sense of suffocation; and extreme restlessness and distress. "Venous congestions supervene in some instances; in others, the patient loses consciousness, and sinks into coma, (Bouillaud.)

*Physical Signs.*—M. Legroux was the first to note that when the concretions embarrassed the play of the valves, or obstructed the orifices, the beats of the heart became dull, smothered and obscure;* and in some instances

---

* M. Legroux attributes the obscurity of the sounds of the heart to the accumulation of blood in the cavities of this organ, (Diss. cit., p. 40.) M. Bouillaud thinks otherwise. "The obscurity of the sounds proceeds from the play of the valves being impeded," (Hope.)

even morbid sounds, as the *bruit de souffle*, &c., were perceived. MM. Bouillaud and Grisolle have since corroborated this, by their own researches.

"When, in a patient who, till then, had presented regular pulsations of the heart, these suddenly become so anomalous, confused, and obscure, that they can no longer be analyzed, we may suspect the formation of a polypous concretion. If the disordered action exists on one side of the heart only, we may consider the thing as almost certain. For instance, if we find the pulsations of the heart, under the sternum, confused and tumultuous, although the day before they had been regular, we may look upon the formation of a polypus in the right cavities as very probable; and the more so, if the contractions of the left ventricle, explored between the fifth and sixth ribs, are more distinct," (Laennec, p. 655.) In polypi of considerable magnitude, "the beats of the heart are irregular, tumultuous, precipitate, and the dullness of the precordial region is more complete and extensive," (Grisolle. Path. Int., p. 353.) "The diminution or the loss of sound in one or more of the cavities of the heart is a certain sign of fibrinous concretions," (Legroux. Diss. cit., p. 41.

There are a number of cases on record, in which a murmur was found to accompany the sounds. Dr. Brouc, in the Journal Hebdomadaire de Med., details a case in which the sounds of the heart were accompanied by an acute whistling murmur. Dr. Desclaux has reported a case in which there was a whining murmur in the precordial region. Dr. Hope never met with examples of this description, but admits the possibility of such a thing, if the polypus should happen to entangle a valve, while the current thorough the auricles and ventricles, remains tolerably free. Before, however, the murmur can be assumed as a sign of polypus, in a given case, it must be proved, first, that it did not previously exist, and, second, that it is not a result of valvular tumefaction from acute endocarditis," (Hope.) While the irregularity described by Laennec and Grisolle, is a sign of polypus in cases where the action of the heart was previously regular, it does not possess the same value in cases where this previous regularity did not exist, and such cases form a large proportion of the cases in which polypus occurs, (Hope.) Should this irregularity be suddenly aggravated—become unusually "anomalous, confused and obscure"—and if, together with this aggravation, the *general signs* be taken into consideration, the diagnosis, even in these latter cases, may be almost always formed with accuracy, (Hope.)

Dr. Hope thinks polypi formed a considerable period previous to death, are not so easily detected—their depo sition being more gradual. Still, if symptoms of the above kind, both physical and general, come on more rapidly than can be accounted for by the ordinary progress of the disease, or if they are such as the disease could not be supposed capable of producing, there is strong reason to suspect a polypus.* The small *globular* polypi often exist without producing any obstacle to the circulation, or any irregularity of the action of the heart.—In general, however, they are found in those who have been in a moribund state for many days, and sometimes many weeks before death.†

*Diagnosis.*—There is a wide difference of opinion between M. Grisolle and M. Bouillaud relative to the certainty of the diagnosis of polypous concretions. M. Grisolle‡ remarks, " that when the functional disturbances which we have enumerated, become suddenly developed in a subject who was not, at the time, suffering from disease of the heart, we should *suspect* the formation of polypous concretions in the cavities of this organ. M. Bou-

*Hope, p. 487. †Loc. cit. ‡Grisolle. Path. Int., p. 353.

illaud affirms that he was enabled to diagnosticate positively, or state as probable the existence of concretions in twelve out of fourteen cases.

In all cases of disease of the heart, whether acute or chronic, previously unattended by any great difficulty or oppression, of orthopnea and considerable disturbance in the beats of the organ, suddenly supervene, we should suspect the formation of concretions.

There is nothing positive known of the time that concretions commence forming, nor of their duration. There is every reason to believe that a large number of them are absorbed, or removed by the torrent of the circulation; while others again, continuing to increase, induce death. The opinion of Prof. Mott, on this subject, we have already given at some length, and shall not again refer to it.

### ARTICLE V.—Prognosis and Treatment of Fibrinous Coagula of the Heart.

I. The prognosis of fibrinous coagula of the heart, when they are sufficiently voluminous to sensibly embarrass the circulation, is, as may be readily inferred from what we have previously said, most grave; they almost always, sooner or later, terminate in death.

When called upon to express an opinion in such cases, we must always give due consideration to the disease with which the concretions are associated, and of which they are strictly but the speaking accident. The more dangerous the disease is in itself, the less promising should be our prognosis of the concretions which are the consequence.

II. *Treatment.*—"No reliable treatment of fibrinous coagula of the heart is known," (Grisolle.) All treatment must necessarily be uncertain in a disease like this, when it is once completely developed, (Bouillaud. "The treatment is mainly preventive," (Hope.) M. Bouillaud advocates general bloodletting, with great zeal. M. Grisolle esteems it useful. Dr. Hope thinks it dangerous.—"To prevent the formation of concretions of blood *in diseases of the heart*, whose property it is to impede the current of the blood, it is useful to employ bloodletting from time to time, and to dilute the blood, in a manner, by aqueous beverages," (Bouillaud.) "I cannot but protest against the indiscriminate, I had almost said random, manner in which M. Bouillaud advocates bloodletting for the prevention of polypus. * * * * I have seen enough of bloodletting, * * * to know that, in dilatation of the heart, in softening, and in advanced cases of valvular disease, bloodletting will not only fail to prevent polypi, but will actually induce them, * * and, moreover, will favor the supervention of dropsy, exhaust the vital powers, and hurry the case to its fatal termination. Moderate bloodletting may, indeed, be admissible, in the early stages of hypertrophy, even when complicated with valvular disease, but these are not the cases in which polypus is apt to occur," (Hope.) "Bloodletting is likewise the best means that can be employed against concretions of the heart *already formed*. It has succeeded beyond my hopes in a female admitted under my care. * * A prey to the most imminent suffocation, and offering, moreover, the physical signs of polypus, * * she has been bled three times, and is at this moment" [eighteen days after] "in a satisfactory state," (Bouillaud.)

The remarks of Dr. Hope, on the treatment of polypus, are of such rare excellence, that I shall copy them almost entire:

"The best mode, according to my observation, of obviating polypus in advanced cases of organic disease of the heart, is, to keep the patient in a state of the *utmost*

*possible* tranquillity, and in the easiest attainable position, so that the circulation may not become embarrassed from being hurried; to avoid not only nauseants and digitalis, but any other unpalatable remedies which disgust or derange the stomach; to avoid, for the same reason, any but the most simple and digestible articles of diet, and not to introduce much into the stomach at once; for the action of the heart invariably becomes disturbed whenever the stomach is considerably distended either by food or, what is almost as bad, by flatulence, the effect of both being to prevent the descent of the diaphragm, in addition to their influence, through the medium of the nervous system. Though the administration of aqueous drinks, with the view of diminishing the coagulability of the blood by dilution, is plausible in theory, I have generally found it inadmissible in practice beyond a moderate extent, in consequence of the intolerable flatulence which it is apt to generate. Nor must it be forgotten that nature often contradicts the very principle itself; for, while the practitioner is diluting the blood, she is often doing her utmost to get rid of that dilution in the form of dropsy; and that her measures are often the wisest, no one will deny who has observed the great relief to the vascular and respiratory system, which frequently follows a considerable serous infiltration. There can be no doubt, indeed, that dropsy, under these circumstances, is a curative effort of nature.

Such are the negative means of obviating polypus; but there are others of a positive nature, to which the practitioner may resort with advantage. The general surface and especially the extremities should be kept comfortably warm, so as, by diffusing the circulation, to prevent congestion in the heart and great vessels. At the same time, cool, fresh air may be admitted to the head, as this often wonderfully alleviates the craving for breath and consequent restlessness of the patient. On the same principle, the use of the fan is most agreeable. Of medicines, I have found those containing sp. eth. sulph. comp. and ammonia sesquicarbonas the most generally useful—probably because, as diffusible stimulants, they distribute and equalize the circulation. In circumstances of great debility, the addition of more permanent stimulants, wine or brandy, becomes indispensable. When paroxysms of congestion of the heart come on, indicated by unusually confused, irregular action of the organ, with an exceedingly small, weak, irregular pulse and suffocative dyspnea, no remedy affords so much relief as a foot bath up to the knees, at as high a temperature as the patient can bear it. If he cannot move, the same may be accomplished with much less fatigue by wringing a small blanket out of hot water, and surrounding the legs with it up to the knees, discomfort being prevented by enveloping the whole in india-rubber cloth. This may be repeated two, three, or even four times a day, if urgently required, the legs, in the intervals, being kept warm with flannel."

The preparations of soda and potass, exercising a solvent effect on the blood, M. Legroux thinks that the salts of these two bases may be used with advantage. He cites no case, however, in support of this theory. A well known effect of these substances is to render the blood florid out of the body, "and the experience of Dr. Stephens in yellow, and other typhoid fevers, and of many in this country in malignant cholera, render it highly probable that they have some corresponding, or at least salutary, effect on the blood within the vessels," (Hope.) We are acquainted with a number of physicians, of high standing, and among them we may mention that estimable gentleman and skilful practitioner, Prof. Lewis Rogers, who regard the agents in question as possessing solvent properties in a very high degree, which may be turned to good account in the treatment of those diseases in which they are indicated. A favorite remedy with Prof. Rogers is the chlorate of potass. Dr. Stephens gives a combination of this and the carbonates of potass. and soda. I agree entirely with Dr. Hope, however, that "further observation is necessary to prove whether these remedies are calculated to obviate polypus." Still, I should not hesitate to administer them were I called to a case of threatened polypus.

I have thus attempted to draw, from different authorities, their several modes of treatment, when the case is not connected with inflammation. It will be remarked that there are between two of them, at least—Dr. Hope and M. Bouillaud—irreconcileable differences of opinion, particularly in relation to the use of the lancet. It has been our good fortune to witness, during several consecutive winters, the practice of the latter of these very eminent gentlemen, and while we must, in candor, admit that we fall very far short of entirely adopting his views of the universal applicability of the lancet to almost every variety of disease, we feel it due to him to state that we have seen, under circumstances like those which now engage us, the very happiest, and, we may almost add, astonishing effects follow free and repeated venesection. It is not our purpose, however, to discuss the merits of this question. We leave that to older and abler hands.

Before concluding our remarks, which have already greatly exceeded our original design, I wish to refer to another state in which the treatment of fibrinous coagula of the heart becomes of even greater interest than in the preceding. It is this: when the disease is associated with inflammation. When this is the case, the inflammation itself must be treated. If the inflammation be of any of the thoracic or abdominal viscera, or, indeed, of any organ or portion of the body, the treatment is to be regulated according to the intensity of the attack, the importance of the suffering organ, its relations to the disease in question, &c., &c.

I now close, with the concluding paragraph in the chapter devoted to this subject, in the treatise of Dr. Hope, to which I am so largely indebted for much contained in this memoir; "and if," says he, "the measures which have been already recommended* for pericarditis and endocarditis be adopted, I believe, according to my own observation, that polypus will be of very rare occurrence."

Louisville, Ky., April 30, 1850.

* These measures are sufficiently well known, to need no repetition here.

